# Four-Week Consumption of Malaysian Honey Reduces Excess Weight Gain and Improves Obesity-Related Parameters in High Fat Diet Induced Obese Rats

**DOI:** 10.1155/2017/1342150

**Published:** 2017-01-26

**Authors:** Suhana Samat, Francis Kanyan Enchang, Fuzina Nor Hussein, Wan Iryani Wan Ismail

**Affiliations:** ^1^Faculty of Pharmacy, Universiti Teknologi MARA (UiTM), Puncak Alam Campus, 42300 Bandar Puncak Alam, Selangor, Malaysia; ^2^Clinical BioPharmaceutics Research Group (CBRG), Pharmaceutical Sciences Core, UiTM, 40450 Shah Alam, Selangor, Malaysia; ^3^Faculty of Veterinary Medicine, Universiti Putra Malaysia (UPM), 43400 Serdang, Selangor, Malaysia; ^4^School of Fundamental Science, Universiti Malaysia Terengganu (UMT), 21030 Kuala Nerus, Terengganu, Malaysia

## Abstract

Many studies revealed the potential of honey consumption in controlling obesity. However, no study has been conducted using Malaysian honey. In this study, we investigated the efficacy of two local Malaysian honey types: Gelam and Acacia honey in reducing excess weight gain and other parameters related to obesity. The quality of both honey types was determined through physicochemical analysis and contents of phenolic and flavonoid. Male Sprague-Dawley rats were induced to become obese using high fat diet (HFD) prior to introduction with/without honey or orlistat for four weeks. Significant reductions in excess weight gain and adiposity index were observed in rats fed with Gelam honey compared to HFD rats. Moreover, levels of plasma glucose, triglycerides, and cholesterol, plasma leptin and resistin, liver enzymes, renal function test, and relative organ weight in Gelam and Acacia honey treated groups were reduced significantly when compared to rats fed with HFD only. Similar results were also displayed in rats treated with orlistat, but with hepatotoxicity effects. In conclusion, consumption of honey can be used to control obesity by regulating lipid metabolism and appears to be more effective than orlistat.

## 1. Introduction

2.1 billion people worldwide are overweight and obese, regardless of their socioeconomic status reported in 2013 [1]. Thus, both overweight and obesity are identified as a major contributor to other chronic diseases such as diabetes, cardiovascular diseases, and even certain cancers [2]. Various factors are known to lead to this health problem, but ease of accessibility to unhealthy foods has been identified as the major culprit [3].

Honey can be described as one of the functional foods. Besides its natural sweet taste, honey has a low glycemic index and other medicinal properties. Its benefit to health and its usage have been well known to mankind since ancient times and were recorded in medical texts from various civilizations [4, 5]. Findings from many studies also showed the ability of honey in controlling overweight and obesity when consumed orally, thus making it a potential antiobesity agent [6–10]. However, the effect using the local honey is still unknown and to be investigated in this study.

A variety of honey found in Malaysia are diverse, which include both blossom and honeydew honey, due to their tropical climate and being rich in floral sources [11]. Biochemical and pharmacological activities of honey vary, depending on its location, weather and humidity, nectar source, and handling during harvesting and storage [11]. In this study, two types of Malaysian honey were used to observe its effects on high fat diet-induced Sprague-Dawley male rats. Gelam honey, categorised as a blossom honey, was produced by* Apis dorsata*, a wild and native honeybee, from nectars of* Melaleuca cajuputi *trees and harvested from the forest. Meanwhile, Acacia honey is honeydew honey produced by* A. mellifera*, also known as European honeybee, from sugary fluids produced by* Acacia mangium* trees and reared by beekeepers in wooden hives [11].

## 2. Methods

### 2.1. Materials

Catechin, Folin-Ciocalteu's reagent, and gallic acid were purchased from Sigma-Aldrich (St. Louis, MO, USA). Sodium carbonate (Na_2_CO_3_), aluminum chloride (AlCl_3_), sodium nitrite (NaNO_2_), and sodium hydroxide (NaOH) were received from Merck (Darmstadt, Germany). All the chemicals used were of analytical grade. The gastrointestinal lipase inhibitor for obesity, orlistat (XENICAL), was obtained from a local drugstore.

### 2.2. Honey Sample Preparation

Gelam honey was harvested from wild honeybees found in a Gelam forest in Terengganu, Malaysia. The Acacia honey was collected from a bee farm under the Department of Agriculture, Johor, Malaysia. The samples were irradiated with 25 kGy of gamma radiation using the radioactive source cobalt 60 (model JS8900), at the Malaysian Nuclear Agency (MINT), Selangor, Malaysia [12]. The irradiated honey was then kept at 4°C away from direct sunlight and in amber bottles.

### 2.3. Physicochemical Analysis of Honey

The pH of Gelam and Acacia honey was determined according to the method described by the International Honey Commission (IHC) [13]. It was measured using a digital pH meter (HI 98127, Hanna Instruments, Mauritius) which was calibrated at room temperature using buffer solutions [14]. Meanwhile, the acidity of both honey types was determined using the titrimetric method (with 0.1 M sodium hydroxide solution) as described in the IHC method [13].

Moisture content was determined using a refractometer. In general, the refractive index indicates the solid content in a sample of honey. The refractive index of both honey types was measured at ambient temperature using an Atago handheld refractometer (Master Refractometer, Japan) and measurements were further corrected at the standard temperature of 20°C by adding a correction factor of 0.00023/°C. The moisture content was measured in triplicate, and the percentage of moisture content that corresponds to the corrected refractive index was calculated [14].

Ash content was measured according to the Association of Official Analytical Chemists (AOAC, 1990) official method 942.05 [15]. Briefly, two grams of each honey type was placed in a porcelain crucible and weighed. After that, samples were dried in an oven at 110°C for four hours, to remove moisture that would cause foaming during the early stages of ashing. The materials were then ashed in an electrical furnace at 600°C for six hours, followed by cooling in a desiccator and then weighed. The ash content on dry basis was calculated according to the following equation:

(1) Percentage  ash  content  on  dry  basis=C−AB−A×100,

where *A* is weight of the crucible, *B* is weight of crucible and sample after evaporation, and *C* is weight of crucible and sample after ashing.

### 2.4. Determination of Total Phenolic Contents

The concentration of total phenolic contents in the Gelam and Acacia honey samples was estimated using Folin-Ciocalteu method with slight modifications. Gallic acid was used to obtain a standard curve (20, 40, 60, 80, and 100 *μ*g/mL; *r* ^2^ = 0.9336) [16]. The concentration of the phenolic compounds from both honey types was measured in triplicate. The results were reported as the mean ± standard deviation and are expressed as mg of gallic acid equivalents (GAEs) per kg of honey.

### 2.5. Determination of Total Flavonoid Content

The total flavonoid content in Gelam and Acacia honey samples was measured using the colorimetric assay as described in Zhishen et al. (1999) [17]. A calibration curve was obtained using standard solutions of catechin (20, 40, 60, 80, and 100 *μ*g/mL; *r* ^2^ = 0.998). The results were expressed as mg catechin equivalents (CE) per kg of honey.

### 2.6. Animal Husbandry

All animal experiments were carried out under protocols approved by the Research Committee on the Ethical Use of Animals (UITM Care), Reference no. 5/2012. Seven-week-old male Sprague-Dawley rats, with body weight ranging from 200 to 220 g, were obtained from Laboratory Facilities of Animal Management (LAFAM), University Technology MARA (UiTM), Puncak Alam, Selangor, Malaysia. Animals were housed as one rat per cage with a 12-hour light/dark cycle [18].

### 2.7. Obesity Induction and Treatment

Control rats were fed with a normal diet, while obese induced rats were fed with a high fat diet (HFD) to elicit diet-induced obesity for four weeks. Then, the animals were subdivided into five groups consisting of six rats per group for another 4 weeks with or without treatment. The groups with high fat diet fed with Gelam honey (HFDGH) and the group receiving high fat diet fed with Acacia honey (HFDAH) served as treatment groups. Rats fed with high fat diet treated with orlistat (HFDO) served as a positive control group.

### 2.8. Body Weight and Meal Pattern Analysis

The body weight of each rat was recorded once a week and the differences in body weight were recorded. In the meal pattern analysis, the amount of food consumed from each rat was measured weekly by subtracting from the quantity of food supplied initially. Energy efficiency of each rat was calculated at the end of the study [18].

### 2.9. Specimen Collection

Blood was collected from the abdominal aorta of the rat, anesthetised using diethyl ether after fasting for 12 hours on the last day of the 8-week feeding period. The collected blood was processed using a microcentrifuge method, and the serum was stored in a freezer at −80°C. The retroperitoneal, epididymal, and visceral fat pads were removed and weighed [18].

### 2.10. Anthropometrical and Adiposity Index Determinations

The body weight and body length were used to determine the following anthropometrical parameters:

- Body mass index (BMI) = body weight (g)/length^2^ (cm^2^).
- Lee's index = cube root of body weight (g)/nose to anus length (cm).

Adiposity index was determined by the total weight of epididymal, visceral, and retroperitoneal fat divided by body weight × 100 and expressed as adiposity percentage (% AI).

### 2.11. Biochemical Analysis

The blood samples were centrifuged at 4 000 revolutions per minute (rpm) for 15 minutes at 4°C. The clear serum obtained was separated, labelled, and subjected to hepatic function tests, including alkaline phosphate (ALP), aspartate aminotransferase (AST) and alanine transaminase (ALT), renal function test (urea and creatinine), and serum lipid profile (glucose, triglycerides, and total cholesterol). These parameters were determined using an Autoanalyser (ILAB 300 Plus Clinical Chemistry Analyser, Milano, Italy). Serum levels of leptin and resistin were assessed using ELISA kits (USCN Life Science and Technology, Wuhan, China). Rat total adiponectin level was determined using Quantikine ELISA kit (R&D Systems Inc., Minneapolis, MN, USA) according to the instructions provided by the manufacturer.

### 2.12. Relative Organ Weight

The relative organ weight (ROW) of each organ was then calculated according to the following equation:

(2) $$\begin{array}{c} ROW=\frac{\left(absolute\;\;organ\;\;weight\;\left(g\right)\times 100\right)}{body\;\;weight\;\;of\;\;rat\;\;on\;\;sacrifice\;\;day\;\left(g\right)}. \end{array}$$

### 2.13. Histological Evaluation

A comprehensive gross observation was carried out on the internal organs such as the liver, spleen, lung, kidneys, and heart. They were observed for any signs of abnormality and for the presence of lesions owing to any effects of the administration of the treatment and high fat diet intake. The organs were then carefully dissected, cleaned from any fat, and weighed.

Each organ was then preserved in 10% buffered formalin for subsequent histopathological examination. The tissues were embedded in paraffin and then sectioned; the sections were cut at 4-5 microns with the rotary microtone, stained with hematoxylin, and examined microscopically.

### 2.14. Statistical Analysis

Results were expressed as mean ± standard error mean (SEM). The statistical significance was determined by one-way analysis of variance (ANOVA) followed by Tukey's test* post hoc* using SPSS software version 18.0 (SPSS, Chicago, IL, USA). Values with a confidence level of *p* < 0.05 were considered significant.

## 3. Results

pH values and ash contents of Gelam and Acacia honey were almost similar (Table 1). However, free acidity of Gelam honey was lower than Acacia honey. The moisture content of Gelam and Acacia honey was over 20%. Gelam honey appeared significantly higher in total phenolic content and flavonoid content compared to Acacia honey (Table 1).

Weight gain and adiposity index in rats fed with high fat diet were significantly increased compared to control group (Table 2), indicating obesity model was successfully established for four weeks in this study. Similar pattern was observed for rats in other groups prior to initiating the treatment for another four weeks. Rats fed with Gelam honey and treated with orlistat, respectively, but not Acacia honey, exhibited significant reduction in both parameters. However, rats fed with Gelam honey showed significant increase in total food intake when compared to other groups. In addition, the rats consistently demonstrated high energy efficiency compared to the other groups. BMI and Lee's index in the treatment groups were not significantly different compared to HFD group. Interestingly, BMI for rats fed with Gelam honey and orlistat were in between control and HFD groups.

Glucose, triglyceride, and cholesterol of rats in HFD group were significantly higher compared to the control rats (Table 3). Similar patterns were seen in rats fed with Acacia honey, but significantly lower compared to HFD group. Both rats in HFDGH groups were significantly reduced in their glucose, triglyceride, and cholesterol levels compared to rats in HFD group.

Similar observation was recorded in HFD group in the level of adipocytokines (leptin, resistin, and adiponectin) once compared with control group (Table 4). Mixed results were found in all treatment groups, whereby the values fall between control and HFD groups. But generally, the treatment reduced all the adipocytokines level compared to the HFD group.

Most parameters from liver enzymes and renal function test in HFD group increased significantly when compared to the control group except for levels of urea and total protein (Table 5). Rats in the treatment groups showed all the measured parameters, except for total protein, were in between levels of control and HFD groups. Interestingly, all treatment groups showed significant decrease in total protein compared to both control and HFD groups.

Relative organ weight (ROW) of liver, heart, and lungs for rats in HFD group showed a significant increase compared to control rats (Table 6). In this parameter, rats fed with Acacia honey showed more prominent effect, where its ROW showed significant increase compared to rats in HFD group, particularly the liver, heart, and lung.

**Figure 1:**
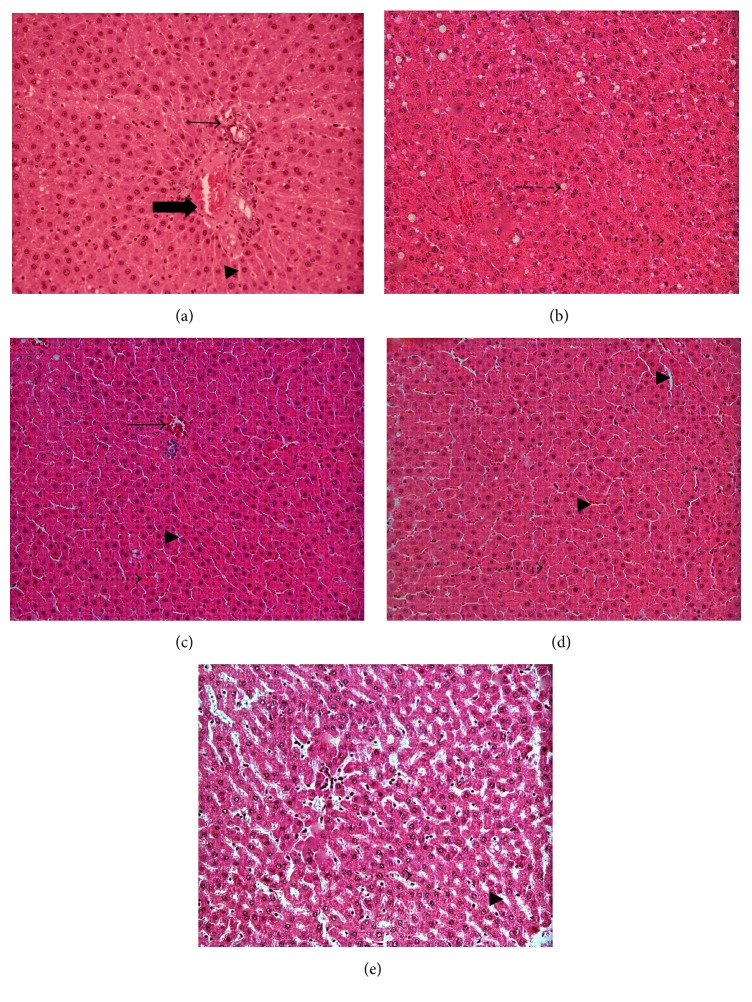
Photomicrographs of rat's liver histology (HE staining ×200) fed with high fat diet for four weeks prior to treatment with Gelam honey, Acacia honey, or orlistat, respectively, for another four weeks. (a) Control (NC); (b) high fat diet (HFD); (c) high fat diet with Gelam honey (HFDGH); (d) high fat diet with Acacia honey (HFDAH); and (e) high fat diet with orlistat (HFDO). Bold arrow: portal vein, normal arrow: central vein, arrow head: sinusoid, dotted arrow: hepatocytes, and dashed arrow: ballooned hepatocytes.

Histopathological evaluations revealed that liver section from rats fed with normal diet (control group) had shown normal morphological appearance (Figure 1(a)). However, livers in the rats that were fed with HFD, fat accumulation, and high number of ballooned hepatocytes were observed (Figure 1(b)). Meanwhile, hepatic tissue of rats fed with both honey types showed normal hepatic tissues with normal hepatic strands; hepatocytes were well arranged from the central vein and were separated by sinusoids (Figures 1(c) and 1(d)). However, the histological architecture of liver sections in rats in HFDO group (Figure 1(e)) showed abnormal patterns, with a mild degree of necrosis and slight lymphocyte infiltration, almost comparable to those of the control group (Figure 1(a)).

## 4. Discussion

Prior to commencing the effect of honey consumption in an obese animal model, physicochemical of Gelam and Acacia honey was measured. The analysis was conducted to standardise the honey condition and to measure the quality of honey used in the study [19, 20]. pH for both honey types was almost similar and within the acceptable range for fresh honey (pH 3.4 to 6.1) set by Codex Standard for honey and the IHC [12, 21, 22]. The pH value was also in parallel to other Malaysian honey reported by Moniruzzaman et al. (2013) [11]. However, Gelam honey was found more acidic compared to Acacia honey. It is because Gelam honey is blossom or floral honey, which contains more antioxidant properties, compared to honeydew honey such as Acacia honey [11].

Antioxidant properties such as total phenolic and flavonoid contents measured in Gelam honey were significantly higher compared to Acacia honey. It contributes to more free acidity in Gelam honey than in Acacia honey as mentioned above. The presence of phenolic acids and flavonoids in honey samples might act as a proton donator leading to the formation of acidified honey with low pH value [23]. This means that phenolic acids and flavonoids are one of the main components responsible for the antioxidant activity of honey that may give a health-protective and therapeutic impact of chronic diseases [24]. Both honey types contain more than 20% of moisture content, slightly higher than level set by the Codex. It is predictable since Malaysia has a tropical climate, which is high in humidity, hot, and rainy all year round [12]. Ash content for both honey types was almost similar. The content depends on the plant nectar and is influenced by several factors including environmental, botanical, and geographical factors [25].

Obesity induction using high fat diet in animal model was used in the study as the approach has high relevancy of mimicking the usual route of obesity occurrence in human [26]. The consumption of high fat diet led to obesity because it facilitates the development of a positive energy balance, leading to an increase in visceral fat deposition, and thus led to abdominal obesity [27]. Seven-week-old male Sprague-Dawley rats, with body weight ranging between 200 and 220 g, were selected for this study, which is a preferred animal model for studying obesity in humans [27, 28]. The rats were induced to become obese for four weeks by monitoring their body weight to increase by more than 125% compared to the rats fed with normal diet [26, 28].

Results showed that, after the first four weeks, followed by the second four-week feeding with high fat diet, rats in HFD group were successfully induced to become obese compared to control rats. Meanwhile, obese induced rats (four weeks) and then fed with honey (four weeks) have shown significant weight reduction compared to HFD group (Table 2). Interesting, rats fed with Gelam honey but not Acacia honey displayed significantly higher total food intake, but low in weight gain compared to HFD group and higher in energy efficiency than displayed by the HFDO group (Table 2). Rationally, a higher kilojoule intake will lead to more weight gain but this was not seen in the rats fed with the honey. The consumption of honey may induce the conversion of excess food into energy instead of being converted into fat for storage.

In addition, rats group fed Gelam honey showed a decrease in adiposity percentage index compared to HFD group (Table 2). Both honey groups (Gelam and Acacia) appeared lower in BMI and Lee's index compared to HFD group. The findings demonstrated that Gelam and Acacia honey are capable of preventing body weight gain, concomitantly helping in maintaining the current body weight. The present study investigated the effects of honey on obese rats and sought to determine whether honey is able to reverse the increased body weight gain caused by high fat diet. Nevertheless, the similarities of these findings strengthen the postulation that honey possesses weight-reducing properties. Result showed that Gelam honey might prevent excessive body weight gain and reduce the body weight increase caused by high fat diet intake.

Similar results had been reported by Chepulis (2009), where rats supplemented with 10% honey* ad libitum* showed a significant reduction in their body weight after 6 weeks of treatment [29]. Clover honey from the United States of America was also reported able to reduce excess weight gain and exhibited antihyperlipidemia* in vivo *[7]. Meanwhile, Romero-Silva et al. (2011) revealed that rats fed with diet containing honey showed significantly reduced adipocyte size after two months of regimen when compared to rats fed sugar [8].

In addition to the results obtained from animal models, data from interventional studies in human subjects supplemented with honey also showed a reduction in body weight, body fat, and a significant reduction in total cholesterol [10]. Another research on overweight subjects done by Yaghoobi et al. (2008) demonstrated that consumption of honey reduced the low-density lipoprotein cholesterol (LDL-C) and triglyceride level and a slight increase in high-density lipoprotein cholesterol (HDL-C) level [10].

Rats in all treatment groups showed significantly lower glucose, triglyceride, and cholesterol levels compared to HFD group. Major composition of honey is mono- and disaccharide sugars, which is glucose, fructose, maltose, and sucrose. However, more than 90% are simple sugars, which are readily absorbed and metabolised after consumption. Usually, not more than 5% sucrose is found in natural blossom honey. Furthermore, hundreds of micronutrients in honey change the way the substance reacts in our digestive system [6]. It is possible that ingestion of honey will reduce plasma glucose level as well, thereby reducing fat deposition [29, 30]. This makes honey have low glycemic index compared to other sweeteners [4].

Moreover, the oral administration of honey significantly decreased the weights of epididymal and retroperitoneal adipose tissues and, ultimately, the total adiposity index of the high fat diet induced obese rats fed with honey. It has been suggested that lower glycemic index of honey may be a good source of quick energy as the calories can be burnt quickly [29, 31]. This may lead to an efficiency in metabolism and decreases amino acid catabolism and fat deposition [29]. Simultaneously, it lowers glucose, triglycerides, and cholesterol level in the bloodstream. Many other studies showed similar findings, as reported by Chepulis (2007); honey fed diet reduced weight gain compared to sucrose [32]. This finding is further strengthened by the previous research done by Nemoseck et al. (2011) and Mushtaq et al. (2011) where they found that honey could reduce weight gain, adiposity, and related biomarkers (leptin, insulin, and adiponectin) and also better blood profiles [7, 31–34]. Another factor that may contribute to a decrease in weight gain is the presence of hydrogen peroxide in honey. Hydrogen peroxide can be a strong insulin mimetic agent [35]. Hydrogen peroxide in honey is produced from the oxidation of glucose by glucose oxidase enzyme.

Adiponectin, leptin, and resistin are adipocytokines that are related to obesity. Leptin is a fat-derived key regulator of appetite and energy expenditure, where its concentration in the plasma is associated with general adiposity [36]. The reductions of leptin and resistin levels in serum level of rats fed with Gelam honey and orlistat reflect a decrease in fat mass but not in HFD (Table 4). This is because hypothalamus receives direct input from hormones, specifically, leptin which crosses the blood-brain barrier and provides information on the levels of peripheral adipose mass [37]. There were predictable changes in neural activity, in brain areas known to be involved in the regulatory, emotional, and cognitive control of food intake, where many of them were reversed by leptin [37]. Following adipocytes loss, leptin decreases in response to visual food cues to hypothalamus [38]. The results from the present study suggested that honey causes significant adipocytes loss (Table 2), indicated by the reduced leptin and resistin level, but an increase in adiponectin level reduced the excess weight gain in the rats (Table 4).

Meanwhile, the biochemical indices such as enzymes found in the liver and kidneys are useful markers for assessment of tissue damage [39]. Tissue enzymes can also indicate tissue cellular damage caused by chemical compounds before structural damage that can be detected by conventional histological techniques [40]. The observation showed that liver enzymes such as ALP, AST, and ALT were significantly higher in HFD and HFDO groups (Table 4). However, for kidney, reductions in urea and total protein level were observed. The abnormality observed indicated liver and kidney problems originated from feeding high fat diet and administration of the chemical compound (orlistat) [41]. There was a significantly higher acid phosphatase activity in the liver of rats fed with HFD and treated with orlistat in HFDO group (Table 6). It may be a result of damage to the lysosomal membrane and consequent leakage of the enzymes from the lysosome into the extracellular fluid [42]. This is an indication of diminished synthetic function of the liver which may consequently lead to enhanced retention of fluid in the tissues spaces and causes organs damage [41].

Above-mentioned results were proven by histological evaluation, where it showed slightly damaged liver tissues in high fat diet (Figure 1(b)) and orlistat (Figure 1(e)). As previously reported by Rao and Hua (2014), fat diet caused mobilization of free fatty acid from adipose tissue and the transportation into hepatocytes [39]. However, in Gelam honey and Acacia honey group it was shown that the levels of liver enzymes and kidney enzymes were within the range [43–45]. The decrease in the level of creatinine, ALP, and ALT activities was a clear indication that honey showed hepatoprotective and renal-protective properties and this is in accordance with the fact that consumption of honey conferred the aforementioned effects [46, 47].

Furthermore, histopathological evaluation for the control rat's liver exhibited normal morphological appearance. It was observed that the hepatic parenchyma consisted of several hepatic lobules separated from each other by very delicate connective tissue septa housing the portal triad (Figure 1(a)). Each hepatic lobule contained a thin walled central vein surrounded by hepatic cords radiating towards the periphery. The hepatic cords were separated from each other by the hepatic sinusoids. The latter appeared wide irregular blood spaces lined by endothelial cells and Kupffer cells. Rats fed with Gelam or Acacia honey displayed similar results with the control rats with no abnormalities in hepatocytes. The hepatocytes were arranged in a trabecula running radiantly from the central vein and were separated by sinusoids containing Kupffer cells (Figures 1(c) and 1(d)). These results were in agreement with previous studies showing that daily consumption of honey gave positive effects on liver function enzymes [46, 47]. However, liver from experimental HFD groups showed lipid accumulation in hepatocytes, abundance of cytoplasm vacuolization, and ballooned hepatocytes while HFDO group showed severe lipid accumulation in hepatocytes (Figure 1(b)). Cytoplasm vacuolization in parenchymatous cells of the liver and hypertrophied hepatocytes were seen in both high fat diet (Figure 1(b)) and orlistat groups (Figure 1(e)). Nevertheless, liver from HFDO groups showed the degenerated hepatocytes and necrosis (Figure 1(e)). The results showed the protective effect of honey compared to toxicity effects of orlistat, in addition to their potential in reducing excess weight gain and obesity.

## 5. Conclusion

Honey is a great natural sweetener and medicinal food, which is rich in nutrients and health benefits. This study recapitulates the effects of honey on obese induced rats. The onset of hepatic steatosis in this model resulted from an increased dietary intake of fat, in addition to excess caloric intake. Moreover, we demonstrated that the daily supplementation of honey might reverse the formation of hepatic steatosis. Furthermore, the present investigation proved that Gelam honey possesses lipid lowering and antioxidative effects in obese induced rats, as well as its weight-reducing ability compared to Acacia honey. However, both honey types showed better effects compared to orlistat, a drug used to control obesity. Due to the promising effects of honey in diet-induced obesity, further investigation is important in order to determine the active compounds in honey followed by identifying the probable mechanisms of action of honey in reducing plasma lipid and its weight maintenance property.

## Figures and Tables

**Table 1:** Physiochemical analysis and total phenolic and total flavonoid contents of Gelam and Acacia honey.

Samples	pH	Free acidity (meq/kg)	Moisture content (%)	Ash (%)	Total phenolic content (GAE mg/L)	Total flavonoid content (CEmg/L)
Gelam honey	3.40 ± 0.02	59.00 ± 1.02	24.90 ± 0.05	0.14 ± 0.01	55.28 ± 0.40	79.57 ± 2.73
Acacia honey	3.55 ± 0.03	85.00 ± 1.71^a^	22.30 ± 1.03	0.18 ± 0.01	33.72 ± 0.45^a^	27.84 ± 0.08^a^

Results are presented as means ± SEM, *n* = 3.

^a^Values are statistically significant at *p* < 0.05 compared to Gelam honey.

**Table 2:** Effect of four-week treatment after four-week obesity inducement in male Sprague-Dawley rats on percentage weight gain, energy efficiency, adiposity index, BMI, and Lee's index.

Test	Group				
	NC	HFD	HFDGH	HFDAH	HFDO
Weight gain (%)	20.32 ± 2.09	44.34 ± 2.15^a^	38.39 ± 2.55^a,b^	40.06 ± 2.40^a^	33.37 ± 1.48^a,b^
Total food intake (kg)	1.23 ± 0.08	1.27 ± 0.11	1.46 ± 0.07^a,b^	1.27 ± 0.10	1.27 ± 0.10
Energy efficiency	0.17 ± 0.03	0.22 ± 0.02^a^	0.26 ± 0.02^a,b^	0.24 ± 0.02^a^	0.18 ± 0.01^b^
Adiposity index (%)	0.60 ± 0.08	1.13 ± 0.04^a^	0.85 ± 0.03^b^	0.96 ± 0.02^b^	0.80 ± 0.12^b^
BMI	0.70 ± 0.01	0.77 ± 0.03^a^	0.74 ± 0.04	0.75 ± 0.06	0.72 ± 0.06
Lee's index	0.30 ± 0.01	0.35 ± 0.01	0.31 ± 0.01	0.31 ± 0.01	0.30 ± 0.01

Results are presented as means ± SEM, *n* = 6.

Values are statistically significant at *p* < 0.05.

^a^Significantly different compared to control (NC) group; ^b^significantly different from high fat diet (HFD) group.

NC: normal control, HFD: high fat diet, HFDGH: high fat diet rats fed with Gelam honey, HFDAH: high fat diet rats fed with Acacia honey, HFDO: high fat diet rats treated with orlistat.

**Table 3:** Effects of four-week treatment after four-week obesity inducement in male Sprague-Dawley rats on levels of glucose, triglyceride, and cholesterol.

Test	Group				
	NC	HFD	HFDGH	HFDAH	HFDO
Glucose (mmol/L)	4.03 ± 0.40	13.00 ± 1.21^a^	5.72 ± 0.44^b^	7.55 ± 0.70^a,b^	5.53 ± 0.51^b^
Triglyceride (mmol/L)	11.10 ± 0.57	17.45 ± 1.10^a^	11.75 ± 1.19^b^	13.61 ± 1.06^b^	14.02 ± 1.01^a,b^
Cholesterol (mmol/L)	5.57 ± 0.37	14.13 ± 0.76^a^	6.60 ± 0.64^b^	8.73 ± 0.82^a,b^	8.53 ± 0.66^a,b^

Results are presented as means ± SEM, *n* = 6.

Values are statistically significant at *p* < 0.05.

^a^Significantly different compared to control (NC) group; ^b^significantly different from high fat diet (HFD) group.

NC: normal control, HFD: high fat diet, HFDGH: high fat diet rats fed with Gelam honey, HFDAH: high fat diet rats fed with Acacia honey, HFDO: high fat diet rats treated with orlistat.

**Table 4:** Effects of four-week treatment after four-week obesity inducement in male Sprague-Dawley rats on adipocytokines levels of leptin, resistin, and adiponectin.

Test	Group				
	NC	HFD	HFDGH	HFDAH	HFDO
Leptin (ng/mL)	8.95 ± 0.76	21.70 ± 1.52^a^	13.66 ± 0.95^a,b^	12.13 ± 1.23^a,b^	13.32 ± 1.04^a,b^
Resistin (ng/mL)	39.21 ± 1.09	48.39 ± 1.7^a^	41.87 ± 1.13^b^	42.18 ± 1.28^b^	41.57 ± 1.03
Adiponectin (ng/mL)	33.67 ± 1.09	30.51 ± 0.78^a^	34.94 ± 1.12^b^	32.78 ± 1.20	41.57 ± 0.91^a,b^

Results are presented as means ± SEM, *n* = 6.

Values are statistically significant at *p* < 0.05.

^a^Significantly different compared to control (NC) group; ^b^significantly different from high fat diet (HFD) group.

NC: normal control, HFD: high fat diet, HFDGH: high fat diet rats fed with Gelam honey, HFDAH: high fat diet rats fed with Acacia honey, HFDO: high fat diet rats treated with orlistat.

**Table 5:** Effects of four weeks of Gelam honey and Acacia honey consumption on levels of liver enzymes and renal function test in rats fed high fat diet for four weeks.

Test	Group				
	NC	HFD	HFDGH	HFDAH	HFDO
ALP (UI)	118.33 ± 5.16	181.50 ± 6.42^a^	135.17 ± 5.35^a,b^	147.00 ± 4.56^a,b^	136.83 ± 3.48^a,b^
AST (U/L)	119.90 ± 1.91	160.40 ± 3.01^a^	120.23 ± 3.05^b^	138.57 ± 2.56^a,b^	130.90 ± 1.08^a,b^
ALT (U/L)	67.53 ± 1.55	81.13 ± 1.60^a^	69.31 ± 1.31^b^	73.13 ± 1.96^b^	71.97 ± 0.62^b^
Urea (mmol/L)	29.75 ± 0.92	16.55 ± 0.84^a^	18.28 ± 0.78^a^	18.41 ± 1.26^a^	17.45 ± 0.95^a^
Creatinine (*μ*mol/L)	20.48 ± 5.12	31.15 ± 6.32^a^	24.15 ± 2.18^a,b^	28.98 ± 4.17^a^	34.82 ± 7.02^a^
Total protein (g/L)	25.35 ± 1.40	23.66 ± 2.38	16.43 ± 1.37^a,b^	17.32 ± 2.82^a,b^	18.49 ± 1.38^a,b^

Results are presented as means ± SEM, *n* = 6.

Values are statistically significant at *p* < 0.05.

^a^Significantly different compared to control (NC) group; ^b^significantly different from high fat diet (HFD) group.

NC: control rats, NC: normal control, HFD: high fat diet, HFDGH: high fat diet rats fed with Gelam honey, HFDAH: high fat diet rats fed with Acacia honey, HFDO: high fat diet rats treated with orlistat, ALP: alkaline phosphate, AST: aspartate aminotransferase, and ALT: alanine aminotransferase.

**Table 6:** Relative organ weights of rats' organs treated for four weeks after fed four-week high fat diet.

Test	Group				
	NC	HFD	HFDGH	HFDAH	HFDO
Liver	2.47 ± 0.43	3.26 ± 0.54^a^	2.75 ± 0.14^b^	2.56 ± 0.22^b^	2.77 ± 0.34^b^
Kidney	0.57 ± 0.06	0.61 ± 0.04	0.56 ± 0.05	0.57 ± 0.04	0.60 ± 0.06
Spleen	0.20 ± 0.03	0.17 ± 0.01	0.16 ± 0.03	0.15 ± 0.03	0.17 ± 0.02
Heart	0.29 ± 0.02	0.33 ± 0.04^a^	0.30 ± 0.01	0.29 ± 0.03^b^	0.30 ± 0.04
Lung	0.35 ± 0.05	0.39 ± 0.02^a^	0.35 ± 0.02^b^	0.34 ± 0.04^b^	0.35 ± 0.05^b^

Results are presented as means ± SEM, *n* = 6.

Values are statistically significant at *p* < 0.05.

^a^Significantly different compared to control (NC) group; ^b^significantly different from high fat diet (HFD) group.

NC: normal control, HFD: high fat diet, HFDGH: high fat diet rats fed with Gelam honey, HFDAH: high fat diet rats fed with Acacia honey, HFDO: high fat diet rats treated with orlistat.
